# Supplementary material for: Effectiveness and Safety of Ayurvedic Medicines in Type 2 Diabetes Mellitus Management: A Systematic Review and Meta-Analysis
Source: Front Pharmacol. 2022 Jun 8;13:821810. doi: 10.3389/fphar.2022.821810 (PMC9213670; doi:10.3389/fphar.2022.821810)
Supplement: Supplementary file 1 [file DataSheet1.zip › Supplementary Material/Forest and Funnel Plots/Zingiber officinale/Zingiber officinale.pdf]

Outcomes	Number of studies	Mean Diff. with 95% CI	Heterogeneity (%)
HbA1c	9	-0.27 [-0.62, 0.07]	68.58
FBG	8	-8.00 [-17.22, 1.23]	0.00
Fasting insulin*	5	-0.86 [-2.11, 0.39]	80.34
Insulin resistance	5	-0.52 [-0.99, -0.04]	68.50
Body weight	5	0.81 [-1.21, 2.83]	0.00
BMI	7	0.61 [0.23, 0.98]	0.00
Waist circumference	4	1.14 [-1.31, 3.58]	9.01
SBP	5	1.37 [-3.90, 6.64]	82.55
DBP	5	0.33 [-2.72, 3.38]	79.78
TC	7	4.84 [-16.93, 26.61]	88.55
HDL-C	7	-2.01 [-4.92, 0.90]	71.38
LDL-C	7	-2.73 [-13.74, 8.29]	74.49
TG	7	-8.48 [-23.70, 6.73]	30.06

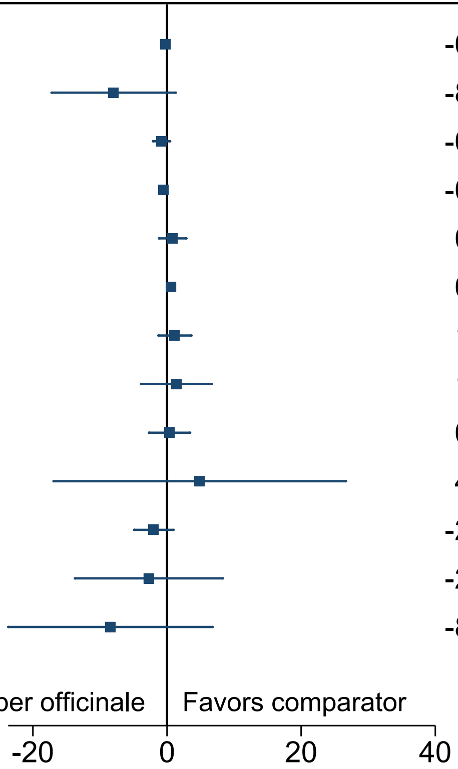

*Favors intervention/comparator as insulin sensitizer
